# The Effects of Angry Expressions and Fearful Expressions on Duration Perception: An ERP Study

**DOI:** 10.3389/fpsyg.2021.570497

**Published:** 2021-06-03

**Authors:** Huazhan Yin, Xiaobing Cui, Youling Bai, Gege Cao, Li Zhang, Yuhong Ou, Dan Li, Jinping Liu

**Affiliations:** ^1^Cognition and Human Behavior Key Laboratory of Hunan Province, Hunan Normal University, Changsha, China; ^2^School of Education Science, Nantong University, Nantong, China; ^3^Hunan Provincial Key Laboratory of Intelligent Computing and Language Information Processing, Hunan Normal University, Changsha, China

**Keywords:** threat-related, time perception, anger, fear, event-related potential

## Abstract

Little is known about the electrophysiological basis of the effect of threat-related emotional stimuli with different motivational direction on duration perception. Thus, event-related potentials were employed to examine the effects of angry expressions and fearful expressions on perception of different duration (490–910 ms). Behavioral results showed there was a greater underestimation of the duration of angry expressions (approach-motivated negative stimuli) than fearful expressions (withdrawal-motivated negative stimuli), compared with neutral expressions. Event-related potentials results showed that, the area of Contingent Negative Variation (CNV) evoked by angry expression, fearful expression and neutral expression gradually increased. These results indicated that specific electrophysiological mechanisms may underlie the attention effects of angry and fearful expressions on timing. Specifically, compared with neutral expressions, fearful expressions and angry expressions both are likely to distract more attentional resources from timer, in particular, angry expressions attract more attention resources than fearful expressions from timer. The major contribution of the current study is to provide electrophysiological evidences of fear vs. anger divergence in the aspect of time perception and to demonstrate beyond the behavioral level that the categorization of threat-related emotions should be refined so to highlight the adaptability of the human defense system.

## Introduction

Timing is a basic ability of human beings that enables us to adapt to the objective environment (Wittmann, 2009). When facing threat-related negative stimuli, human beings would change subjective timing to adapt to the objective environment (Droit-Volet and Gil, 2009). For example, when individual encounters threat stimuli (e.g., snakes, spiders, etc.), perceived duration is extended so that the individual has enough time to take the next action. At present, although researchers have known about the effect of threat-related negative emotional stimulus on the perception of time, however, little is known about the effect of different subtypes of threat-related negative stimuli on duration processing (Lake et al., 2016). Thus, the present study intends to continue to explore this question. It not only lays the foundation for revealing the influence of threat-related emotion on temporal perception, but also helps people to make better use of emotional timing rules to adapt to the environment.

The pacemaker-accumulator (PA) model, as one of the classical theoretical models explaining timing process (Gibbon et al., 1984), postulates the existence of an internal pacemaker that emits pulses with a certain frequency to an accumulator. There may be a switch manipulated by attention resources controlling the transmission of pulses (Meck, 1984; LeDoux, 2012). More precisely, pulses are passed into the accumulator when one pays attention to timing, and pulses are blocked when one does not pay attention to timing. According to previous studies, there are three kinds of mechanism of threat-related emotional stimuli on duration perception (Schirmer, 2011). Firstly, increasing arousal leads to overestimation of duration. Secondly, emotional stimulation can induce attention orientation, and then lead to the change of latency of timing switch, which finally lead to overestimation of duration. Third, when the emotional stimulus is a timing signal, attention to emotional stimulus will lead to overestimation of duration, and when the emotional stimulus is a distractor, the duration will be shortened. In short, arousal and attention are two important sources of variation affecting timing (Gibbon, 1977; Gibbon et al., 1984).

It is more and more recognized that threat-related emotions should be examined separately because each discrete emotion may have a specific function and consequently a specific attention-related effect on duration perception (Frijda, 1988; Vaish et al., 2008; Droit-Volet and Gil, 2009; Krusemark and Li, 2011). However, direct comparison of duration perception between different threat-related emotions remains very few (Tipples, 2008). Fear refers to the bad feeling that put oneself in worry about impending danger and intensely urge to protect oneself to get out of that situation (Vaish et al., 2008), while anger represents a collection of certain kinds of stimuli that forecast dangers (such as an unfulfilled wish or frustrated behavior) in the environment, which could bring about physical and psychological harm to the individual (Lerner and Keltner, 2000). Previous research has proved that physiological and behavioral responses and cognitive processes induced by fearful and angry stimuli particularly were divergent: the pattern of heart rate increasing caused by anger and fear is similar, but the peripheral vascular function of anger and fear is different (Ekman et al., 1983; Levenson, 1992). Since fearful stimuli promote with-drawal behavior, and angry stimuli promote approach behavior (Hutcherson and Gross, 2011), and the theory motivational dimension of emotions hold that the intensity of the motivation of emotional stimulation plays a key role in the allocation of attention resources (Bradley et al., 2001). Thus, the present study was designed to examine the attention-related time distortions caused by these two subtypes of threat-related emotional stimuli. In consideration of arousal and attention are two important sources of variation affecting timing (Gibbon, 1977; Gibbon et al., 1984), and some studies found that the arousal of the emotional expression may also influence timing (Droit-Volet and Meck, 2007; Stetson et al., 2007; Grommet et al., 2011; Lambrechts et al., 2011). Therefore, the arousal of the threat-related negative emotional stimuli in this study was carefully controlled so as to prevent our results from being contaminated by the arousal differences among different emotional stimuli conditions. Furthermore, since the contributions of attention and arousal are often difficult to disentangle at the behavioral level, the event-related potentials (ERPs) were used to search for the neural correlates of the different underlying processes about influencing on duration perception by angry and fearful stimuli. To this day, a few studies have explored the effect of emotion stimuli on temporal perception using ERP technology. For example, one study found that the average amplitude of contingent negative variation (CNV) induced by emotional conditions (anger and happy) was lower than that induced by neutral condition. In addition, under the emotional conditions, the P160 and P240 amplitudes were enhanced and the N230 amplitude was decreased (Gan et al., 2009). These findings suggest that temporal processing can be modulated by emotion stimulus, even within 200 ms of the stimulus onset, and that the attentional bias for emotion stimulus attenuates the cognitive resources for time perception. Other study directly investigated the less frequently considered role of attention as an alternative mediator of these effects with ERPs. In this study participants were asked to produce short intervals (0.9, 1.5, 2.7, and 3.3 s) while viewing high arousal images with pleasant and unpleasant contents in comparison to neutral images. The valence-specificity of affective attention revealed by ERPs combined with behavioral timing results suggested that attention processes indeed contribute to emotion-induced temporal distortions, especially for longer target intervals (Tamm et al., 2014). Morever, there’s another study also explored the different effects between two threat-associated emotions (fear and disgust) on duration perception (Zhang et al., 2014). These findings indicated that specific neural mechanisms may underlie the attention effects of different subtypes of threat-related emotions on timing, compared with neutral faces, fearful faces are likely to attract more attentional resources while disgusted faces may attract less attentional resources for emotional processing. In view of the dimension of emotional motivation, fearful expressions and angry expressions were different motivational direction (approaching or withdrawing) of emotional stimuli, which can infer that the attention-related mechanisms of time perception aroused by fear and anger are distinct, resulting in distinct ERP patterns when participants are asked to estimate the duration presented by fearful and angry expressions. The present study was to record the ERPs changes in the course of temporal generalization task and emotional stimuli with arousal controlled was employed to verify how fear and anger affect duration perception.

According to previous literature (Macar and Vidal, 2004; Zhang et al., 2014), we analyzed three ERP components: vertex positive potential (VPP), N170, and CNV. VPP is a large positive ERP with a latency of 170 ms and elicited at fronto-central electrodes (Jeffreys, 1996), and N170 is a large negative ERP with a latency of 170 ms and elicited at lateral posterior electrodes (Bentin et al., 1996). The main feature of these two scalp components is their enhanced response to faces compared to other multiple object categories (e.g., Bentin et al., 1996; Jeffreys, 1996; Rossion et al., 2003). Many studies have also proposed that N170, coordinated with emotions, created less amplitudes elicited in reaction to neutral facial expressions than emotional facial expressions, which correspond to its positive counterpart component of VPP (Batty and Taylor, 2003; Blau et al., 2007; Schyns et al., 2007). More importantly, numerous studies have also demonstrated that there was a positive correlation between the average amplitude of CNV and length of the estimated duration (Macar and Vidal, 2003) and the area of CNV was a positive correlation with the attentional resources of timer in different emotional sitmulus conditions (Zhang et al., 2014). Therefore, It is expected that early ERP components such as N170 and VPP would be enhanced by emotions, and that the area amplitudes of the slow CNV component may display separated waveforms in fear and anger conditions, indicating specific neural mechanisms underlying the attention effects of different subtypes of threat-related emotions on duration perception.

## Methods

### Participants

Through G-power3.1 software (alpha level 0.05, power 0.8, effect size 0.3, subject group 1) (Faul et al., 2007), the total number of participants was calculated as 24. Thus, a total of 30 undergraduate students (16 female, 14 male; mean age = 21.9 years, range = 18–23) taken part in the experiment are from Southwest Normal University, Chongqing, China. All participants had normal or corrected-to-normal vision and had no prior neurological/psychiatric history. The study received written consent from every participant who was paid for their participation. This study was reviewed and approved by the local ethics committee of Southwest Normal University.

### Stimuli

The experiment included a learning stage and a testing stage. A white oval was used as the stimulus of “target” duration for the learning phase. During the testing phase, 60 black-and-white photos (20 angry, 20 fearful, and 20 neutral expressions) consisting of the same number of facial images for males and females from the native Chinese Facial Affective Picture System (CFAPS) were used as the stimulus of “probe” duration and presented at the center of computer monitoring (Gong et al., 2011). The valence and arousal score of each expression was asked to evaluate on a nine-point scale, and analyzed to see if the two types of negative expressions were significantly different from the neutral expressions. The main effect of the expression type was present [*F*(2,38) = 45.468, *p* < 0.001, η^2^*p* = 0.705; mean ± standard deviation: angry = 3.10 ± 0.50, fearful = 2.95 ± 0.45, neutral = 4.21 ± 0.53; angry/fearful vs. neutral: *ps* < 0.001] in valence, while no significant difference among the arousal scores of the three types of expressions [*F*(2,38) = 0.116, *p* = 0.841, η^2^*p* = 0.006; angry = 5.18 ± 1.32, fearful = 5.30 ± 0.82, neutral = 5.17 ± 0.44; angry vs. neutral: *p* = 0.964; fearful vs. neutral: *p* = 0.527] occurred. Note that the chosen 20 neutral expressions have a generally higher arousal scores in comparison with the rest of the neutral expressions in the CFAPS to prohibit our results from being polluted by difference in arousal among emotional states [there was a sum of 422 neutral expressions in the CFAPS (valence = 4.29 ± 0.52; arousal = 3.84 ± 0.69)].

### Procedure

**FIGURE 1:**
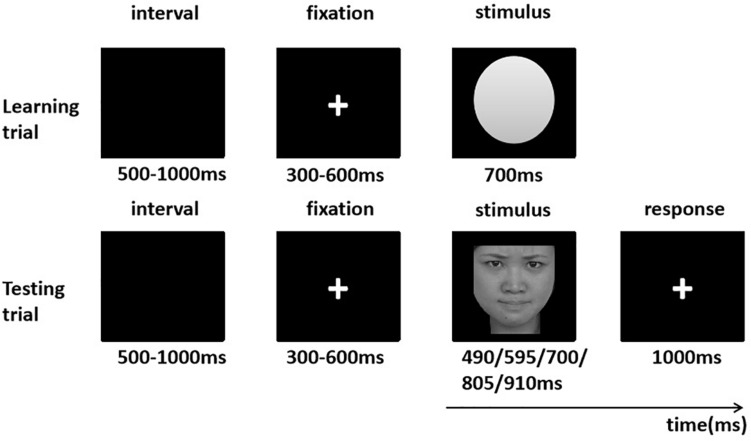
Procedure of events in a sample learning and testing trial. The face picture was selected from the native Chinese Facial Affective Picture System (CFAPS) (Gong et al., 2011).

After visiting the laboratory, each participant was seated about 70 cm in front of a DELL computer in a special experimental room. The computer (19-inch CRT monitor, refresh rate 100 Hz) was responsible for the presentation of the experimental stimuli and recording the participants’ response data with E-Prime 2.0 (Psychology Software Tools, Inc., Pittsburgh, PA). Each stimulus was the same as each other in contrast and brightness (4.0° × 4.6° visual angle) against a black background. A temporal generalization task was employed to explore the timing mechanism (Pouthas et al., 2000; Macar and Vidal, 2004; Gil and Droit-Volet, 2011a,b). The task included a learning stage and a testing stage (Figure 1). We asked the participants to match a probe duration (equivalent to or not equivalent to) with a previously presented target duration. During the learning stage, participants were required to view a white oval that was the same size as the emotional images for the target duration (700 ms) 10 times on end. During the testing phase, participants were exposed to angry, fearful, or neutral pictures for the probe duration (490, 595, 700, 805, and 910 ms) (Pouthas et al., 2000). In each trial, the participant had to press the “D” or “K” button (yes or no) on the computer keyboard to judge whether the probe duration was “the same” as the target duration or not. The testing stage was comprised of 12 blocks. Each block included 100 trials. Each probe duration (490, 595, 700, 805, and 910 ms) included 20 trials in each block. Between blocks is the balance between subjects, and the trails within blocks are random. In order to eliminate the interference between emotional stimuli, each block only contained one kind of trials to induce one emotion. Morever, to prohibit the participants from forgetting the target duration, it was presented six times in succession before the beginning of each block. The break between blocks was decided by self-termination of participants. In each trial, participants whose response latency was less than 100 ms or more than 1,500 ms were considered unavailable.

### EEG Recording and Analysis

Recordings were made with a sampling frequency of 500 Hz, an amplifier bandpass of 0.05–80 Hz (6 dB/octave), and an electrode impedance 5 kΩ. Eye movements were subtracted (Semlitsch et al., 1986). Tin electrodes, mounted with the reference electrode on the left and right mastoids, were used to record electrical brain activity from 64 scalp sites in an elastic cap (Brain Products GmbH, Gilching, Germany). Trials with EOG artifacts (mean EOG voltage exceeding ± 150 μV) and those contaminated with artifacts were excluded from averaging because of amplifier clipping, bursts of electromyographic activity, or peak-to-peak deflection exceeding ±150 μV. The data analysis and result revealed in this study were designed in Matlab R2014a (MathWorks, Natick, United States). In the present study, we primarily analyzed ERP produced by three facial expressions (anger, fear, and neutral) under five probe duration conditions and epochs changed after the onset of facial expressions within 1,200 ms from the baseline pre-stimulus at 200 ms. On the basis of the relevant literature (Monfort et al., 2000; Pouthas et al., 2000), the mean amplitudes of fronto-central VPP, occipito-temporal N170, and the area amplitudes of the CNV-like component across different sets of electrodes were analyzed. Moreover, the electrode sites and time windows for each effect were chosen in line with grand-mean ERP topographies. About the VPP, three sites of FC1, FCz, and FC2 (time window = 170–190 ms) were figured. For the N170, four sites of the mean amplitudes of P7, P8, PO7, and PO8 (time window = 170–190 ms) were counted. For the CNV, three sites of the mean area amplitudes of FC1, FCz, and FC2 were counted. The CNV area amplitude is based on the integral part of the ERP waveform below the two zero intersections of the time axis (Macar and Vidal, 2003; Zhang et al., 2014).

### Statistics

SPSS software 20.0 (IBM, Somers, United States) was used for statistical analysis. Mean ± standard deviation are represented as descriptive data. A repeated measures ANOVA with a *post hoc* analysis was performed on behavioral data with affective category (angry, fearful, and neutral expressions) and probe duration (490, 595, 700, 805, and 910 ms) as factors. The significance level was set at 0.05. Where appropriate, the Greenhouse-Geisser correction was applied to ANOVA tests. *Post hoc* testing of significant main effects was conducted using Bonferroni method. A simple effects model was employed to explain the significant interactions. Partial eta-squared (η^2^*_*p*_*) was recorded to prove the effect size in ANOVA tests, with 0.0099 showing a small effect, 0.0588 displaying a medium effect, and 0.1379 indexing a large effect (Richardson, 2011). For assessing the Behavioral and ERP preformance of temporal generalization task, a Percentage of “yes” responses and response time, three affect-sensitive components were identified in stimulus-locked ERPs: VPP, N170, and CNV were calculated (Macar and Vidal, 2003).

‘

## Results

### Behaviors

#### Percentage of “Yes” Responses

**FIGURE 2:**
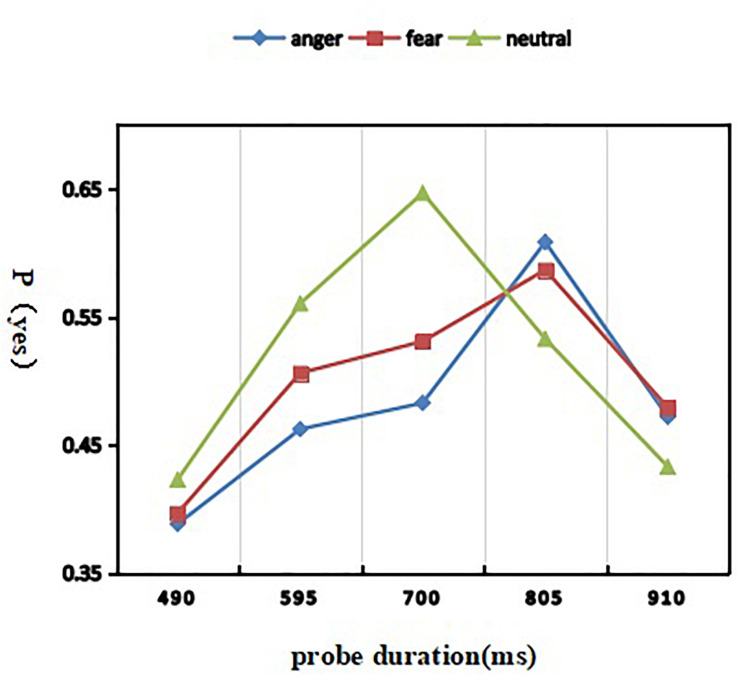
Mean proportion of “yes” responses as a function of emotion and duration conditions.

A 3 (discrete emotion: anger, fear, and neutral) × 5 (duration: 490, 595, 700, 805, and 910 ms) repeated-measures ANOVA was used to analyze the proportion of “yes” responses (Figure 2). The interaction effect of discrete emotion by probe duration was highly present [*F*(8, 232) = 34.240; *p* < 0.001; η^2^*_*p*_* = 0.541], meaning that the temporal distortion induced by two threat-related emotional stimuli were dependent on the probe duration. Simple effect analysis showed that the proportion of “same” responses was modulated by emotion in 490–[*F*(2, 58) = 5.341; *p* = 0.007; η^2^*_*p*_* = 0.156], 595–[*F*(2, 58) = 30.119; *p* < 0.001; η^2^*_*p*_* = 0.509], 700–[*F*(2, 58) = 84.282; *p* < 0.001; η^2^*_*p*_* = 0.744], 805–[*F*(2, 58) = 30.368; *p* < 0.001; η^2^*_*p*_* = 0.512], 910–[*F*(2, 58) = 6.800; *p* = 0.002; η^2^*_*p*_* = 0.190] ms. In particular, 46.2% angry, 50.6% fearful, and 56.0% neutral expressions under 595 ms condition were estimated as having a duration of 700 ms, while 60.8% angry, 58.7% fearful, and 53.3% neutral expressions under 805 ms condition were estimated as having a duration of 700 ms. The pattern proportion of “yes” responses showed that, compared with neutral expression, the duration of fearful expression and angry expression were estimated to be shorter. Morever, a main effect of probe duration was significant [*F*(4, 116) = 110.939; *p* < 0.001; η^2^*_*p*_* = 0.793], indicating that the proportion of “yes” responses was smaller in the 490 ms (*M* = 0.403 ± 0.021) and 910 ms conditions (*M* = 0.461 ± 0.023), compared to the 595 ms (*M* = 0.510 ± 0.026), 700 ms (*M* = 0.553 ± 0.032), and 805 ms conditions (*M* = 0.576 ± 0.034) (*ps* < 0.001). The other main effect of emotional expression was present [*F*(2, 58) = 25.680; *p* < 0.001; η^2^*_*p*_* = 0.470]. *Post hoc* analyses revealed that, compared with the duration of neutral expression (*M* = 0.519 ± 0.025), the duration of angry expression (*M* = 0.483 ± 0.025) was more underestimation than that of fear expression (*M* = 0.500 ± 0.023).

#### Response Time (RT)

**FIGURE 3:**
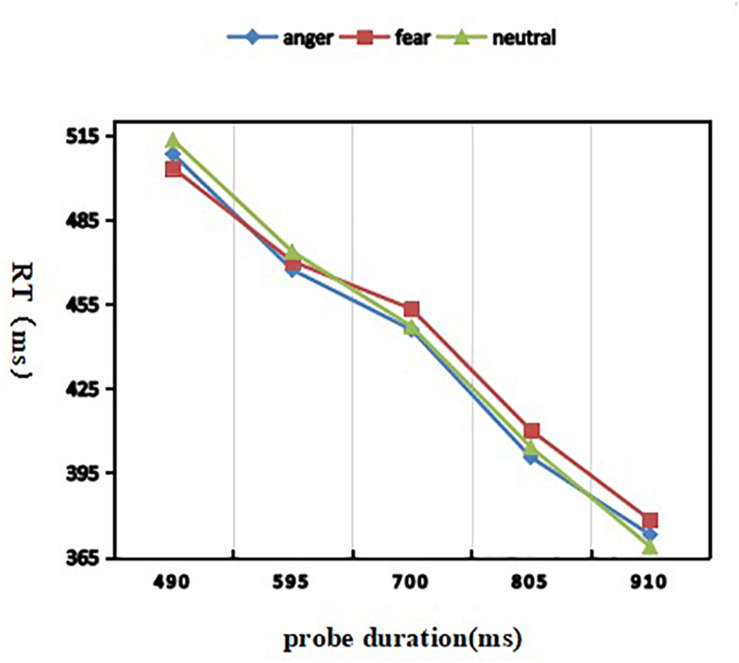
Mean reaction time as a function of emotion and duration conditions.

A 3 (discrete emotion: anger, fear, vs. neutral) × 5 (duration: 490, 595, 700, 805, vs. 910 ms) repeated-measures ANOVA was used to analyze the response time (Figure 3). There was a significant main effect of probe duration [*F*(4, 116) = 146.630; *p* <0.001; η^2^*_*p*_* = 0.835)]. *Post hoc* analyses revealed that, the RT went down with probe duration (M_490_ = 507 ± 46 ms, M595 = 469 ± 41 ms, M_700_ = 448 ± 38 ms, M_805_ = 404 ± 32 ms, M_910_ = 373 ± 30 ms in 490, 595, 700, 805, and 910 ms conditions) going on. Both the effect of discrete emotion [*F*(2,58) = 0.167; *p* = 0.846] and the interaction effect of discrete emotion by probe duration failed to reach significance [*F*(8, 232) = 0.195; *p* = 0.991].

#### Stimulus-Locked Affective ERP Components

Table 1 indicates descriptive statistical results of affective effects in all three stimulus-locked components (VPP, N170, and CNV).

**TABLE 1:** Event-related potentials VPP, N170, and CNV for different experimental stimuli across the nrobe durations.

**Probe duration (ms)**		**Angry expression**	**Fearful expression**	
	490 ms	5.032 (0.043)^**#^	4.542 (0.038)**	4.204 (0.032)
	595 ms	5.121 (0.050)^**#^	4.562 (0.021)**	4.267 (0.036)
VPP	700 ms	4.984 (0.052)^**#^	4.613 (0.032)**	4.229 (0.049)
	805 ms	5.038 (0.048)^**#^	4.564 (0.037)**	4.174 (0.039)
	910 ms	4.999 (0.056)^**#^	4.571 (0.042)**	4.242 (0.036)
	490 ms	−4.088 (0.043)^**#^	−3.409 (0.038)**	−3.146 (0.032)
	595 ms	−3.999 (0.050)^**#^	−3.388 (0.021)**	−3.083 (0.036)
N170	700 ms	−4.136 (0.052)^**#^	−3.337 (0.032)**	−3.121 (0.049)
	805 ms	−4.082 (0.048)^**#^	−3.386 (0.037)**	−3.176 (0.039)
	910 ms	−4.121 (0.056)^**#^	−3.379 (0.042)**	−3.108 (0.036)
	490 ms	1.879 (0.035)^**#^	2.082 (0.048)**	2.249 (0.031)
	595 ms	2.101 (0.063)^**#^	2.405 (0.059)**	2.737 (0.050)
CNV	700 ms	2.258 (0.052)^**#^	2.55 (0.045)**	2.986 (0.036)
	805 ms	2.397 (0.063)^**#^	3.070 (0.069)**	3.476 (0.046)
	910 ms	2.544 (0.044)^**#^	3.063 (0.062)**	3.581 (0.079)

**p < 0.001; *p < 0.01; significant difference from the Neutral stimuli; ^#^p < 0.001; significant difference from the fearful stimuli. VPP (μV); N170 (μV); and CNV (μV⋅S); mean values with standard deviations.

##### VPP

**FIGURE 4:**
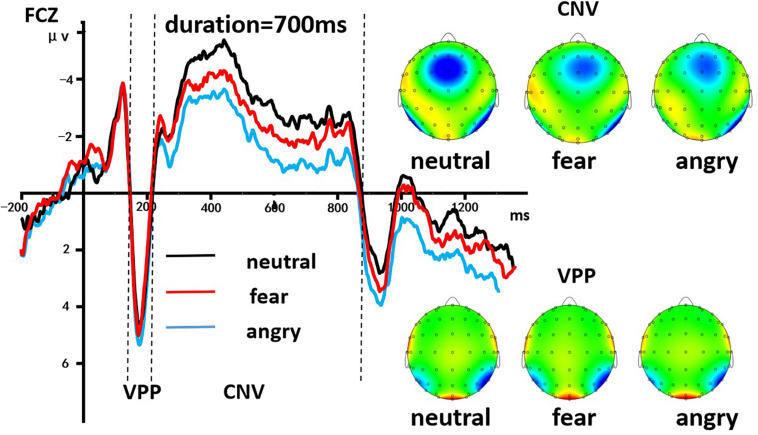
The grand-mean ERP waveforms of the VPP and the CNV components. The CNV area amplitude was based on the integral part of the ERP waveform below the two zero intersections of the time axis. Mean amplitudes of the VPP component were calculated during 170–190 ms. The black line indicated neutral emotion, the red line indicated fear emotion, and the blue line indicated angry emotion.

A 3 (discrete emotion: anger, fear, vs. neutral) × 5 (duration: 490, 595, 700, 805, vs. 910 ms) × 2 (hemisphere: left, right) repeated-measures ANOVA was employed to analyze the average amplitudes of the VPP component. There was a significant main effect of discrete emotion was present, [*F*(2, 58) = 442.244, *p* < 0.001, η^2^*_*p*_* = 0.938], indicating that the VPP amplitude induced by neutral expressions (*M* = 4.223 ± 0.035 μV), fearful expressions (*M* = 4.570 ± 0.039 μV), and angry expressions (*M* = 5.035 ± 0.044 μV) gradually increased significantly (*ps* < 0.001). Morever, the main effect of the hemisphere also was significant [*F*(1, 29) = 63.158, *p* < 0.001, η^2^*_*p*_* = 0.685], indicating that the average amplitudes of the VPP in the left hemisphere (*M* = 4.577 ± 0.040 μV) were significantly smaller than the right hemisphere (*M* = 4.642 ± 0.041 μV). However, the other effects were not significant (*p* > 0.05, Figure 4).

##### N170

**FIGURE 5:**
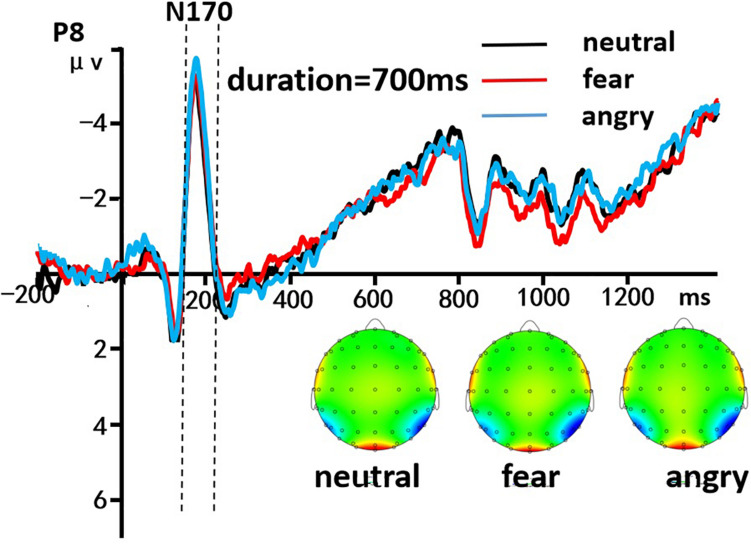
The grand-mean ERP waveforms of the N170 component. Mean amplitudes of the N170 component were calculated during 170–190 ms. The black line indicated neutral emotion, the red line indicated fear emotion, and the blue line indicated angry emotion.

A 3 (discrete emotion: anger, fear, vs. neutral) × 5 (duration: 490, 595, 700, 805, vs. 910 ms) × 2 (hemisphere: left, right) repeated-measures ANOVA was used to analyze the average amplitudes of N170 component. There was a significant main effect of discrete emotion was present, [*F*(2, 58) = 588.714, *p* < 0.001, η^2^*_*p*_* = 0.953], indicating that the N170 amplitude induced by neutral expressions (*M* = -3.690 ± 0.032 μV), fearful expressions (*M* = -3.942 ± 0.036 μV), and angry expressions (*M* = -4.645 ± 0.042 μV) gradually increased significantly (*ps* < 0.001). Morever, The main effect of the hemisphere was significant [*F*(1, 29) = 11,691.782, *p* < 0.001, η^2^*_*p*_* = 0.998], indicating that the average amplitudes of the N170 in the left hemisphere (*M* = -3.568 ± 0.030 μV) were significantly smaller than the right hemisphere (*M* = -4.617 ± 0.043 μV). However, the other effect were not significant (*p* > 0.05, Figure 5).

##### CNV

A 3 (discrete emotion: anger, fear, vs. neutral) × 5 (duration: 490, 595, 700, 805, vs. 910 ms) × 2 (hemisphere: left, right) repeated-measures ANOVA was used to analyze the average amplitudes of the CNV area. There was a significant main effect of discrete emotion, [*F*(2, 58) = 198.651, *p* < 0.001, η^2^*_*p*_* = 0.873], indicating the CNV area that induced by neutral expressions (*M* = 3.006 ± 0.028 μV⋅s), fearful expressions (*M* = 2.634 ± 0.025 μV⋅s), and angry expressions (*M* = 2.236 ± 0.022 μV⋅s) gradually decreased significantly (*ps* < 0.001). The main effect of the hemisphere was also significant [*F*(1,29) = 23,684.294, *p* < 0.001, η^2^*p* = 0.999], indicating that the average amplitudes of the CNV area in the left hemisphere (*M* = 2.190 μV⋅s) were significantly smaller than the right hemisphere (*M* = 3.060 ± 0.029 μ V⋅s). In addition, The main effect of probe duration was also significant, [*F*(4, 116) = 325.321, *p* < 0.001, η^2^*_*p*_* = 0.918], with *post hoc* analyses indicating that the CNV area for 490 ms (*M* = 2.070 ± 0.019 μV⋅s) was smaller than for 595 ms (*M* = 2.414 ± 0.023 μV⋅s); the CNV area for 595 ms was smaller than for 700 ms (*M* = 2.598 ± 0.026 μV⋅s). However, there was no difference between the area amplitude of CNV at 700, 805 (*M* = 2.981 ± 0.028 μV⋅s), and 910 ms (*M* = 3.062 ± 0.031 μV⋅s). The interaction effect of discrete emotion × duration was significant [*F*(8, 232) = 7.457, *p* < 0.001, η^2^*p* = 0.205]. Simple effect analysis indicated that the effect of emotion on the CNV area first increased, then decreased with the increasing duration of stimulus presentation [*F*(2, 58) = 19.235; *p* < 0.001; η^2^*_*p*_* = 0.399] in 490-ms condition; [*F*(2, 58) = 25.514; *p* < 0.001; η^2^*_*p*_* = 0.468] in 595-ms condition; [*F*(2, 58) = 58.789; *p* < 0.001; η^2^*_*p*_* = 0.670] in 700-ms condition; [*F*(2, 58) = 71.132; *p* < 0.001; η^2^*_*p*_* = 0.710] in 805 ms condition; [*F*(2, 58) = 64.360; *p* < 0.001; η^2^*_*p*_* = 0.689] in 910-ms condition (Figure 4).

## Discussion

The main aim of the current study was to investigate the effects of two threat-related emotional stimulus, which are arousal-controlled angry expressions and fearful expressions on duration perception with ERPs. Behavioral results revealed that compared with neutral expression, fearful expression and angry expression were being estimated shorter. Several stimulus-locked ERP components were used to objectively characterize the states generated by affective expressions. Accordingly, the ERP data revealed that the early stage of emotion modulated duration perception was represented by larger fronto-central VPP amplitudes and occipito-temporal N170 amplitudes in fear/angry expression conditions, compared with neutral condition. Morever, the CNV, which is an online index of timing, displayed separated waveforms in different emotional stimuli conditions, with smaller amplitudes in angry and fearful conditions when compared with neutral condition.

The behavioral data in this study indicated that the underestimation of angry expression and fearful expression was remarkable, because previous behavioral studies often found that the duration of emotional expression was overestimated compared with neutral expression (Gil et al., 2007; Tipples, 2008; Gil and Droit-Volet, 2011a; Fayolle et al., 2015). According to the PA model, the distort of duration perception is often explained in terms of arousal-induced and attention-related mechanisms (Matell and Meck, 2000; Droit-Volet et al., 2004; Droit-Volet and Meck, 2007; Wittmann and Paulus, 2008). The mechanism of arousal-induced supposed that the internal clock was accelerated theoretically by the physiological arousal level of emotional stimuli, leading to a great number of accumulated pulses, thus bringing about an overestimation of duration perception (Droit-Volet et al., 2004; Noulhiane et al., 2007). After controlling the arousal ratings across the three emotional conditions, this study mainly investigated the attention-related time distortions caused by fearful and angry expression. It was hypothesized that, compared with neutral expression, fearful expression and angry expression attract more attention to the emotional content of the stimuli and thus divert processing resources from the timer, resulting in an underestimation of time. Consistent with this hypothesis, the behavioral data showed that the duration of fearful expression and angry expression were judged shorter than that of neutral expression. Similarly, there were another two behavioral studies controlled the arousal levels of negative pictures (mainly fearful ones, e.g., spiders or rats) when investigating the effect of emotion on timing, which found that negative pictures were overestimated in high-arousal condition (arousal range = 6.5–7.5) while they were underestimated in low-arousal condition (arousal range = 4–5.7), compared with low-arousal neutral pictures (Angrilli et al., 1997). This opposite effect of negative pictures as a function of arousal suggested that the attention-related mechanism mainly works for low arousing stimuli whereas the arousal-induced mechanism mainly works for high arousing stimuli (Angrilli et al., 1997; Buhusi and Meck, 2006; Droit-Volet and Meck, 2007). In this study, the arousal of emotional expression is just a low arousal condition (arousal scores in this study: angry expressions = 5.18 ± 1.32; fearful expressions = 5.30 ± 0.82). Thus, we investigated in this study *per se* relatively low arousing stimuli of fear and anger so as to explore the attention-related mechanism of duration perception. In addition, there might be another explanation of motivational direction influencing the perception of duration (Gable et al., 2017). Anger arises in situations where approach toward a goal is interrupted or an anticipated reward is blocked (Carver and Scheier, 2008), and that approach motivation in negative affective states related to the perception of time shortening. However, the underestimation of the duration of fear expression was not consistent with previous studies (e.g., Gable et al., 2017). The study found that a withdrawal-motivated negative state (disgust) caused the perception of time to lengthen relative to a neutral state, and fear belongs to a type of withdrawal-motivated negative state. Therefore, the effect of motivational direction of emotional stimulation on duration perception is still a problem that needs to be further explored.

In addition, a particularly noteworthy finding was that the duration estimation of angry expression was shorter than that of fear expression. It has been discussed in the literature that fearful and angry expressions could evoke different physiological responses and cognitive processes. Anger evokes similar patterns with injections of epinephrine and nor-epinephrine combined, and the patterns evoked by fear and injections of epinephrine are similar. The interconnection of anger to physiological variables was significantly higher than in the case of fear, showing that it is integrated more during anger (AX, 1953). This may mean that angry expression stimuli attract more attention resources from the time dimension of emotional stimuli than fearful expression stimuli, which leads to more underestimation.

The ERP data in this study found that larger fronto-central VPP amplitudes in fearful/angry expression conditions than in the neutral condition were represented in the early stage of emotion-modulated time perception. Many studies have compared emotional and neutral expressions which has alike arousal but higher ratings in emotional expressions (Batty and Taylor, 2003; Williams, 2006; Blau et al., 2007), albeit VPP was proves to be regulated by emotional expressions for smaller amplitudes in neutral expressions than in emotional expressions (such as fear) (Williams, 2006; Schyns et al., 2007). A natural covariation entre arousal and the valence grades of emotional stimuli occurred in the manipulation (i.e., a U-shaped connection in valence-arousal coordinate). In these studies, the effects of emotional-controlled found on ERP components could be caused by diversification of values or changes in arousal, or both. Accordingly, this study also discovered that the amplitudes of VPP could be raised by the valence of facial expressions separately. Fortunately, the occipito-temporal N170 amplitudes are also modulated by emotional expressions. In line with the early structural encoding operations described by Bruce and Young (1986), early ERP components (e.g., N170) are thought to reflect rapid structural encoding of faces. This may mean that the structure of emotional expressions is more complex than that of neutral expressions. Furthermore, the most novel ERP finding of this study is that the main effect of emotion was significant, suggesting that the CNV area in the neutral condition (*M* = 3.006 μV⋅s) was greater than that in the fear condition (*M* = 2.634 μV⋅s) and angry condition (*M* = 2.236 μV⋅s). The medial fronto-central cortex (typically at the electrode site of FCz) before the supplementary motor area (SMA) was considered to be the neurosubstrate of the timing function reflected in the CNV (Macar and Vidal, 2004). The SMA showed consistent activation in temporal processing (Coull et al., 2008). Furthermore, the vitality of the SMA was heightened by diverting attention to timing optionally (Coull et al., 2008). On the basis of such thesis, it can be known to all that the degree of attention turned to timing determined the level of neural activation that contributes to time perception (Macar and Vidal, 2004). As a result, compared with neutral expressions, a smaller CNV area was observed in the fearful condition and was more apparent in the angry condition. This indicated that emotional processing occupied more attentional resources, resulting in underestimation of time. Therefore, compared with CNV results, despite both fearful and angry expressions being threat-related, different attention deviation effects were observed in the two emotional conditions. The current study contributed different neural correlates between angry expressions and fearful expressions on duration perception, and demonstrates that it cannot focus on behavioral aspects but refine the classification of threat-associated emotions to stress the fitness of the human defense system (Krusemark and Li, 2011).

Finally, a non-linearity was displayed in the interaction effect of emotion by stimulus duration on the CNV area: with the growing duration of stimuli, the effect of emotion increased at first but then decreased. Thus, the effect of emotion on the perception of time appears to be greater in the medium durations (595, 700, and 805 ms) than in the excessive durations (490 and 910 ms). This CNV pattern which showed the duration from 300 to 800 ms was the highest sensitivity on time perception is congruent with previous findings. The main effect of stimulus duration was also significant. The *post hoc* analyses indicated that the CNV area for 490 ms was smaller than for 595 ms, which is smaller than for 700 ms; however, there is no difference between the area amplitude of CNV at 805 and 910 ms. This result is consistent with that of previous research (Pfeuty et al., 2005). The CNV results implied that the attentional mechanism could not interpret the pattern of all data. It is necessary to conduct further researches to inspect more factors that influence temporal perception. In particular, one of the major shortcomings of this study is that it did not monitor the subjects’ basic conditions such as depression, anxiety and mood, which may pollute the results of this study.

## Conclusion

In summary, arousal-controlled emotional stimuli was used in the current study to verify and compare the effect of two subtypes of threat-related emotional expression on duration perception. We suggested that angry and fearful expressions had different effects on the attention-associated mechanism of time perception, which is issued in divergent behavioral and ERP patterns. Particularly, there was a greater underestimation of the timing of angry expressions than fearful expressions when compared with neutral expressions. ERP results showed that, compared with neutral expressions, a smaller CNV area was observed in the fearful expression condition and angry expression condition, and the trend was more apparent in the angry expression condition. We propose that it is necessary to refine the categorization of threat-related emotions in order to emphasize the adaptability of human defense systems to optimize actions in response to various environmental hazards.

## Data Availability Statement

The raw data supporting the conclusions of this article will be made available by the authors, without undue reservation.

## Ethics Statement

The studies involving human participants were reviewed and approved by the Ethics Committee Southwest Normal University. The patients/participants provided their written informed consent to participate in this study.

## Author Contributions

HY, YB, DL, XC, and JL designed and coordinated the study. YB, GC, LZ, XC, and YO carried out experiments and data process. GC drafted the manuscript. HY reviewed the manuscript. All authors gave the final approval for publication.

## Conflict of Interest

The authors declare that the research was conducted in the absence of any commercial or financial relationships that could be construed as a potential conflict of interest.
